# Effects of Chronic Ethanol Consumption and Ovariectomy on the Spontaneous Alveolar Bone Loss in Rats

**DOI:** 10.1155/2020/8873462

**Published:** 2020-11-12

**Authors:** Priscila Cunha Nascimento, Leonardo Oliveira Bittencourt, Soraya O. Pinto, Luana N. S. Santana, Renata Duarte Souza-Rodrigues, Armando L. Pereira-Neto, Cristiane S. F. Maia, Cassiano K. Rösing, Rafael Rodrigues Lima

**Affiliations:** ^1^Laboratory of Functional and Structural Biology, Institute of Biological Sciences, Federal University of Pará, Belém-Pará 66075-110, Brazil; ^2^School of Dentistry, Institute of Health Sciences, Federal University of Pará, Belém-Pará 66075-110, Brazil; ^3^Laboratory Pharmacology of Inflammation and Behavior, Institute of Health Sciences, Federal University of Pará, Belém-Pará 66075-110, Brazil; ^4^Department of Periodontology, Faculty of Dentistry, Federal University of Rio Grande do Sul, Porto Alegre 90040-060, Brazil

## Abstract

Postmenopausal estrogen deficiency and ethanol (EtOH) abuse are known risk factors for different diseases including bone tissues. However, little is known about the synergic effects of EtOH abuse and estrogen deficiency on alveolar bone loss in women. The present study evaluated the effects of EtOH chronic exposure and ovariectomy on the alveolar bone loss in female rats. For this, 40 female Wistar rats were randomly divided into 4 groups: control, EtOH exposure, ovariectomy (OVX), and OVX plus EtOH exposure. Initially, half of the animals were ovariectomized at 75 days of age. After that, the groups received distilled water or EtOH 6.5 g/kg/day (20% w/v) for 55 days via gavage. Thereafter, animals were sacrificed and the mandibles were collected, dissected, and separated into hemimandibles. Alveolar bone loss was evaluated by measuring the distance between the cementoenamel junction and the alveolar bone crest through a stereomicroscope in 3 different anatomical regions of the tissue. One-way ANOVA and post hoc Tukey were used to compare groups (*p* < 0.05). The results showed that the ovariectomy and EtOH exposure per se were able to induce alveolar bone loss, and their association did intensify significantly the effect. Therefore, OVX associated with heavy EtOH exposure increase the spontaneous alveolar bone loss in rats.

## 1. Introduction

According to the World Health Organization (WHO), the abusive consumption of ethanol (EtOH) induces around 3.3 million death per year (5.9% of all world deaths) and approximately 5.1% of the morbidities in the world [1]. In recent years, the intake of EtOH has been increased mainly among women [1,2].

The vulnerability of women by the harmful effects of EtOH exposure emerges as an important public health issue since females have increased the EtOH consumption due to changes in the social and economic profile of countries [3,4]. Such vulnerability could be explained by factors such as lower body weight, lower capacity of alcohol metabolization, and a higher proportion of body fat, which contributes to the higher alcohol levels as compared to men after the same dose of EtOH [5].

Among the harmful effects, studies indicate that EtOH chronic consumption can induce osteoporosis due to the alteration of both the production and resorption pathways of bone remodeling [6–9]. Animal models of alcohol-induced bone loss are important tools in the study of pathologic bone loss. A study from our group demonstrated that heavy EtOH exposure induces alveolar bone loss in rats with no other periodontitis-inducing factor (e.g., the presence of ligature) [10].

On the other side, sexual hormones, such as estrogen, modulate skeletal homeostasis through direct effects on osteocytes and osteoblasts, reducing the bone remodeling and supporting the maintenance of bone formation, as well as inducing inhibitory effects by blocking the activation of osteoclasts directly or via osteoblasts and immune cells (T-cells), which reduces bone resorption (for review see Cauley [11]).

The present study aimed to verify the impact of EtOH exposure, associated or not with ovariectomy (OVX) on spontaneous alveolar bone loss in female rats. It was hypothesized that chronic EtOH heavy exposure intensifies spontaneous alveolar bone loss associated with induced estrogen deficiency.

## 2. Materials and Methods

### 2.1. Ethics Statement and Experimental Animals

A total of 40 female 75-day-old Wistar rats, weighing 80–100 g, were used in this investigation. The animals were obtained from the Animal House from Federal University of Pará (UFPA) and maintained in collective cages (with 4 animals each); water and food were available *ad libitum* and kept in a climate-controlled environment (25°C) with 12 : 12°h light/dark cycle (lights on 7:30 AM). All procedures were approved by the Ethics Committee on Experimental Animals of the Federal University of Pará under protocol BIO-042-12 and followed the guidelines suggested by the NIH Guide for the Care and Use of Laboratory Animals [12].

### 2.2. Experimental Groups

The animals were equally and randomly distributed into four groups: Control group, which received distilled water by gavage (H_2_O_dist_) without OVX procedure (Control, *n* = 10); EtOH exposure group, which received 6.5 g/kg/day of EtOH, 22.5% w/v by gavage (EtOH group, *n* = 10); OVX group (at 75^th^ day of age), which received distilled water by gavage (OVX group, *n* = 10); and OVX group which received EtOH as the protocol of group EtOH (EtOH + OVX, *n* = 10). The sample size was defined based on a previous study [13] and calculated by G^∗^Power 3.1 software. For this, a statistical power of 80%, an error of 5%, and predicting a sample loss of 20% were considered at the end of the study.

Firstly, rats from OVX groups were ovariectomized on the first experimental day (75-day-old). For the OVX procedure as previously described [14,15], the animals were anesthetized with a mixture of ketamine hydrochloride (90 mg/kg, i.p.) and xylazine (10 mg/kg, i.p.); in addition, we administered ketoprofen (5 mg/kg. *sc*) 30 minutes prior to the surgery. The surgical access occurred through the medial line of the animal abdomen, near to the kidney's levels, below the last rib, with an incision of 1.5 cm vertically, and both ovaries and surrounding fat tissue were removed. After ovariectomy, muscular and skin layers were sutured. The animals of the control group and EtOH group have realized the surgical access but no ovaries were removed. After the surgery, the rats were allocated in individual cages for a period of seven days to surgery recovery and received analgesic medication for pain relief (ketoprofen, 5 mg/kg, sc) for additional two consecutive days, following the ethic recommendations of postsurgical procedures.

Fifteen days after surgery, all animals started to be exposed to EtOH or H_2_O_dist_ through intragastric gavage for 55 days (i.e., until 145-day-old) according to a procedure previously described [16,17]. The administrated dosage of EtOH was based on previous studies from our group and simulated chronic consumption [18–21]. The animals' body weight was regularly controlled during experimental periods at 75, 90, 120, and 145 days of age to dose adjustment and investigate the possible effects of EtOH administration on the overall poor nutrition levels that may directly affect oral health.

### 2.3. Perfusion and Tissue Procedure

At the 145^th^ day of age, following exposure to EtOH or water, animals were deeply anesthetized with ketamine hydrochloride (90 mg/kg, i.p.) and xylazine hydrochloride (10 mg/kg, i.p.). Then, the intracardiac perfused with heparinized 0.9% saline solution followed by 4% paraformaldehyde in 0.2 M phosphate buffer was realized. Surgical manipulation was performed only after both the corneal and the paw withdraw reflexes were abolished. Jaws were removed, dissected, and separated in hemimandibles.

### 2.4. Measurement of Alveolar Bone Loss

**Figure 1:**
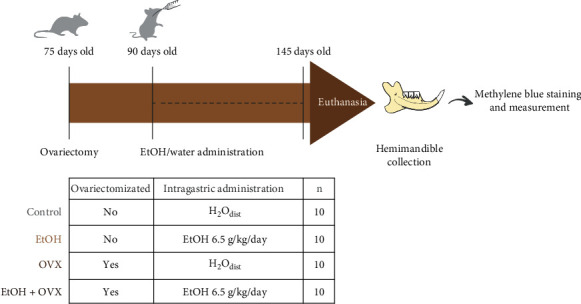
Study design. First, the female Wistar rats were ovariectomized at 75 days of age. After 15 days of the procedure, distilled water–H_2_O_dist_ (Control group; OVX group) or ethanol–EtOH (EtOH group; EtOH + OVX) were administered by intragastric gavage during 55 days. At the end of the administration, the animals (145 days old) were euthanized. Their hemimandibles were collected. Afterwards, they were stained with methylene blue allowing the measurement of alveolar bone loss with stereomicroscope.

The hemimandibles of each group were observed in a stereomicroscope (Discovery V8 Zeiss, Germany). Samples were immersed in 8% sodium hypochlorite (NaOCl) for 4 h after being washed with distilled water in an ultrasonic bath for 10 min. To highlight the cementoenamel junction, the samples were immersed in 1% methylene blue for 60 s, followed by water wash. Then, they were dried at room temperature, prepared (fixed in wax) in which the lingual surface of the jaws was perpendicular to the observation axis, and examined using a reticulum graph attached to the eyepiece stereomicroscope. Images were obtained with a 6.1-megapixel camera (Cannon, Powershot A640) attached to the stereomicroscope (3.2x) that was standardized to give measurements in millimeters. Using the stereomicroscope images, the distance between the cementoenamel junction (CEJ) and the alveolar bone crest (ABC) was measured in the second molar at seven palatal sites of each rat's hemimandible to establish a representative mean per animal [22–24], using the Image J software and the scale obtained by the photomicroscopy system, in order to minimize any type of inaccuracy. The examiner that performed the measurements was unaware of group allocation.  Figure 1 demonstrates the flowchart of the study.

### 2.5. Statistical Analyses

All results were tabulated and expressed as means ± standard error of the mean (SEM) or 95% confidence interval (CI). Then, we performed the Shapiro–Wilk test for normality analyses. The data of the weight curve was evaluated by two-way ANOVA. Statistical comparisons between groups were performed using the one-way ANOVA test. After both tests, the Tukey post hoc test was applied. The data were analyzed by GraphPad Prism 7.0 software (GraphPad Software Inc., La Jolla, CA, USA).

## 3. Results

### 3.1. The EtOH Exposure and/or OVX Procedure Did Not Affect the Body Weight Gain of Rats

**Figure 2:**
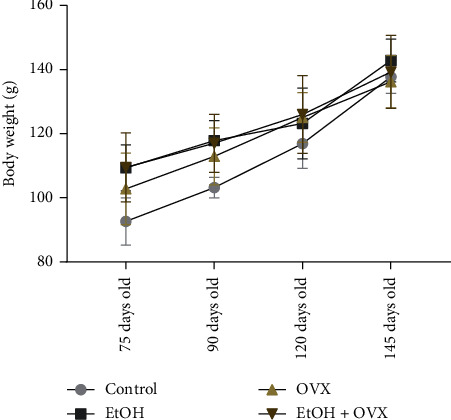
Effects of chronic administration of ethanol (during 55 days) and ovariectomy on the body weight of female Wistar rats (*n* = 10 per group). Results are expressed as mean ± standard error of mean. Two-way ANOVA and Tukey's post hoc test, *p* < 0.05. Ethanol chronic exposure and/or OVX procedure increase/s alveolar bone loss.

During the experimental period no animal died during surgeries and postsurgical periods. The systemic effect of EtOH consumption observed by analyzing animals body weight showed that all groups gained weight when compared to the baseline (Control: 92.7 ± 7.4g; EtOH: 109.5 ± 7.2g; OXV: 102.9 ± 11.2g; EtOH + OVX: 109.65 ± 10.8g) and final body weight (Control: 138 ± 5.17g; EtOH: 143.2 ± 6.7g; OVX: 136.5 ± 8.2g; EtOH + OVX: 139.6 ± 11.4g, *p* < 0.01, Figure 2). At the end of the experimental period, no significant difference was detected between the final weight in the groups receiving ethanol or water distilled and ovariectomized or not (*p* > 0.0001, Figure 2).

### 3.2. EtOH Chronic Exposure and/or OVX procedure increase the alveolar bone loss

**Figure 3:**
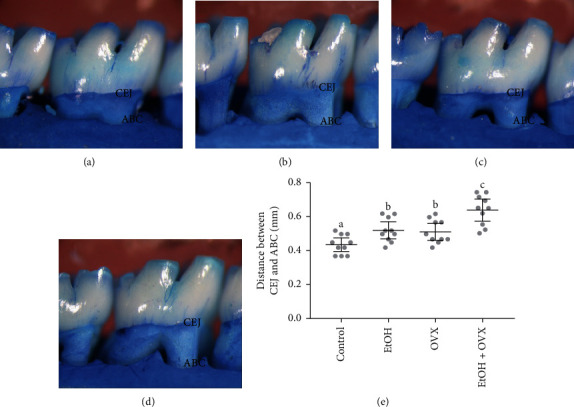
Effects of chronic administration of ethanol (during 55 days) and ovariectomy on the alveolar bone of female Wistar rats (*n* = 10 per group). Representative photomicrographs hemimandibles of the (a) Control group, (b) Ethanol group (EtOH), (c) Ovariectomized group (OVX), and (d) Ovariectomized group and ethanol administration (EtOH + OVX). Results are expressed as mean and 95% CI of the mean of (e) distance between cement-enamel junction (CEJ) and the alveolar bone crest (ABC) in millimeters (mm). One-way ANOVA and Tukey's post hoc test, *p* < 0.05. Similar overwritten letters mean no significant statistical differences.

Illustrative photomicrographs of hemimandibles from the experimental groups are presented in Figure 3. The distance measured between the cementoenamel junction and the alveolar bone crest used as indicative of alveolar bone loss is presented in Figure 3(e). Statistical analyses showed a significant higher bone loss in the EtOH + OVX (0.63 ± 0.01 mm) compared to the control group that received distilled water and was not submitted to OVX (Control: 0.45 ± 0.01 mm, *p* < 0.0001) and higher bone loss in comparison to the EtOH and OVX groups (p<0.05). Moreover, the variables per se induced alveolar bone loss in comparison to control group, butdid not showsignificant difference between them (EtOH: 0.56 ± 0.02 mm; OVX: 0.55 ± 0.02 mm; *p* > 0.05).

During the experimental period no animal died during surgeries and postsurgical periods. The systemic effect of alcohol consumption observed by analyzing animals body weight showed that all groups gained weight when compared to the baseline (Control: 92.7 ± 7.4g; EtOH: 109.5 ± 7.2g; OXV: 102.9 ± 11.2g; EtOH + OVX: 109.65 ± 10.8g) and final body weight (Control: 138 ± 5.17g; EtOH: 143.2 ± 6.7g; OVX: 136.5 ± 8.2g; EtOH + OVX: 139.6 ± 11.4g, *p* < 0.01, Figure 2). At the end of the experimental period, no significant difference was detected between the final weight in the groups receiving ethanol or water distilled and ovariectomized or not (*p* > 0.0001, Figure 2).

3.2. EtOH Chronic Exposure and/or OVX procedure increase the alveolar bone loss Illustrative photomicrographs of hemimandibles from the experimental groups are presented in Figure 3. The distance measured between the cementoenamel junction and the alveolar bone crest used as indicative of alveolar bone loss is presented in Figure 3(e). Statistical analyses showed a significant higher bone loss in the EtOH + OVX (0.63 ± 0.01 mm) compared to the control group that received distilled water and was not submitted to OVX (Control: 0.45 ± 0.01 mm, *p* < 0.0001) and higher bone loss in comparison to the EtOH and OVX groups (p<0.05). Moreover, the variables per se induced alveolar bone loss in comparison to control group, butdid not showsignificant difference between them (EtOH: 0.56 ± 0.02 mm; OVX: 0.55 ± 0.02 mm; *p* > 0.05).

## 4. Discussion

The present study evaluated the impact of OVX and/or exposure to alcohol on spontaneous alveolar bone loss in female Wistar rats. The results demonstrated that loss of periodontal support is facilitated with the exposure to alcohol and after OVX and the combined effect of OVX and exposure to alcohol is even more deleterious.

Considering the fundamental importance of maintaining the integrity of the dentoalveolar complex, that EtOH consumption beyond social levels leads to addiction with a series of negative consequences, and that the hormonal deficiency is frequent among women, in this study we are bringing strong support that there is a synergistic effect to oral health when the combination of EtOH and OVX is present. Both are considered risk indicators for periodontal breakdown, probably participating in the causal chain of periodontal diseases. The current findings demonstrate that chronic EtOH consumption was able to increase alveolar bone loss, by increasing the distance between the cement-enamel junction and alveolar bone provoked by ovariectomy. Similar findings have been demonstrated previously [6,10,25–27]. However, as we hypothesized, the combination of both exposures leads to a synergy among them and is a novel finding in periodontal literature.

It is well described that estrogen deficiency and alcohol exposure are two risk factors for bone loss [10,11,28–31]. In fact, studies claim that heavy and prolonged alcohol intake induces periodontitis and leads to higher degrees of alveolar bone loss. [10,32] The alcohol intake protocol used in the present study mimics the typical pattern of alcohol misuse in alcoholics, especially in an intermittent drinking pattern [33]. According to Livy et al. [34], a daily dose of EtOH starting at 3.8–9 g/kg/day leads to a high blood alcohol concentration (BAC) (up to 200 mg/dL) and BAC up to 100 mg/dL in rats, upon intragastric administration of EtOH. Moderate-to-high binge EtOH exposure consists of BAC 150–225 mg/dL [35], which is similar to the blood levels reached among alcoholics in emergency care (BAC around 250 mg/dL) [36]. In addition, our group has validated this heavy drinking protocol, in which, on the last day of administration, the BAC found was 242.6 ± 14.5 [37]. In this way, although we have not performed BAC determinations due to the toxicokinetic of EtOH characteristic and our experimental design, according to Livy et al. [34] and Da Silva et al.'s [37] studies, our protocol of EtOH exposure should have reached a BAC of approximately 170–250 mg/dL, characterized as heavy EtOH drinking.

Studies from our group demonstrated that heavy binge drinking induces alveolar bone loss in the absence of any other factor inducing periodontal disease in adolescent rats [10]. In the present research, such evidence was observed in adult female rats, in which alveolar bone loss was detected in the absence of periodontitis also. The measurement of spontaneous alveolar bone loss has been considered a sound measurement of the impact of periodontal inflammation. If, on one hand, no induction method was used to generate plaque accumulation such as ligatures, for example, on the other hand, the simple presence of the naturally accumulating dental deposits leads to periodontal breakdown [38].

Moreover, estrogen deficiency plays a main role in osteoporosis and, consequently, may increase the risk for periodontal breakdown [29,32,39]. In a study, Xu et al. [29] demonstrated that ovariectomy-induced estrogen deficiency reduces alveolar crest height and bone formation and negatively affects the trabecular structure. In the same study, several markers of bone resorption serum levels were increased. However, OVX by itself was not able to reduce the alveolar crest height but aggravated the reduction when associated with experimental periodontitis induced by ligatures. In contrast, the present study claims that estrogen deficiency per se was sufficient to display alveolar bone loss, increasing the distance measured between the cementoenamel junction and the alveolar bone crest, without any additional plaque-accumulating device, for example. These results agree with our research, in which ovariectomy, without induction of periodontal breakdown, induced an increase in the distance between the cement-enamel junction and the alveolar bone crest that was characterized as a parameter of alveolar bone loss. Although our study design did not determinate estrogen levels, interestingly, a previous study has pointed that 4 weeks after OVX, the plasma estrogen levels reduced (sham: 9.51 ± 2.17 pg/mL vs. OVX: 5.45 ± 070 pg/mL) [40] and another showed that 9 weeks after OVX, the levels of estrogen is significantly lower (66.1 ± 15.4 pmoles/L) in comparison to sham-operated animals (85.7 ± 14.5 pmoles/L) [41], suggesting that this period of estrogen deficiency may promote the bone impairment observed in our study.

The study conducted by Lee et al. [42] found similar results, although comparing ovariectomized rats and rats treated with lipopolysaccharide. Liu et al. [43] found a correlation between a greater number of osteoclasts along the margins of the buccal alveolar of bone proper samples from OVX rats compared with controls. Expression of OPG and RANKL was significantly higher, and that of DMP1-C was significantly lower, in OVX rats compared with control rats. However, this hypothesis has not been confirmed by other groups [44].

To verify the influence of EtOH intake on alveolar bone loss induced by ovariectomy, a study [31] demonstrated that this association adversely affected the quality of alveolar bone, with changes in the stoichiometry composition of hydroxyapatite in the alveolar bone crest, characterized by reducing calcium/phosphate ratio. Following that, Alonso et al. [45] suggested that chronic ethanol administration associated with estrogen deficiency in rats also produces alveolar bone crest loss and inflammatory process, in periodontal tissues. Thus, both studies affirm that such adverse effects may increase the susceptibility of the host to the progression of periodontal disease. Our results agree with Marchini et al. [31] and Alonso et al.'s [45] studies since heavy chronic alcohol exposure associated with estrogen deficiency presented synergistic effects and increased alveolar bone loss when compared to groups with sole alcohol exposure or ovariectomy.

According to another study of this same group, through micro X-ray fluorescence spectrometry, alcohol intake in the presence of estrogen deficiency modified the stoichiometry composition of hydroxyapatite in the alveolar bone crest, demonstrated as a reduction in the calcium/phosphorus ratios, which, in turn, could affect bone remodeling process [31]. Our results suggest that the increase of alcohol exposure period (in our study, the rats were exposed to alcohol for 55 days) associated with estrogen deficiency induced by OVX could potentialize the alveolar bone loss rather than alcohol and OVX alone since the bone loss was more prominent when the combination was present.

Indeed, the amount of food ingestion seems to be more important in terms of bone quality and bone homeostasis [46]. Proposing to control this variable, our rats received *ad libitum* diet and their weights were verified during the experiment. The results of body weight demonstrated that no statistically significant difference was observed between the final weights in the groups receiving ethanol or water distilled and ovariectomized or not; thus, in our study, the results in terms of bone quality no were influenced by diet.

The present study has strengths and limitations. Among strengths, we emphasize on all the possibilities of controlling for additional variables that a study in animals allows, with the chance to isolate each one of the exposure variables. Also, Wistar rats have demonstrated to have interesting similarity in terms of periodontal disease to humans. The limitations of the study reside in the impossibility of direct translation of the knowledge. However, it gives new information for clinical research.

In conclusion, the present study demonstrated that OVX and chronic EtOH exposure increased the distance between the cementoenamel junction and the alveolar bone crest. Furthermore, based on the results of this experiment, alcohol associated with OVX presents a synergistic effect in the pathogenesis of spontaneous alveolar bone loss in rats. Our results point to the need for further investigation with other methodologies and analyses that better elucidate the damage mechanisms triggered in the alveolar bone through this comorbidity.

## Data Availability

The data used to support the findings of this study are included within the article.
